# The challenges and opportunities of personal health data tracking and sharing amongst people living with HIV in the United Kingdom and their specialist healthcare providers

**DOI:** 10.1177/20552076251383420

**Published:** 2025-09-26

**Authors:** Emily Jay Nicholls, Karen C Lloyd, Katarina Hoernke, Alexander Maddams, Caroline Claisse, Abigail C Durrant, Shema Tariq, Jo Gibbs

**Affiliations:** 1 Institute for Global Health, 4919 University College London, London, UK; 2 Norwich Medical School, University of East Anglia, Norwich, UK; 3 Open Lab, School of Computing, 5994 Newcastle University, Newcastle upon Tyne, UK; 4 4954 Central and North West London NHS Foundation Trust, London, UK

**Keywords:** Personal health data, HIV, long term conditions, clinical care, data tracking, self-monitoring

## Abstract

**Objective:**

There has been an increase in digital tools and wearable devices that can be used by individuals to collect, track and share their personal health data (PHD). Collecting PHD could be particularly useful for those living with long-term conditions such as HIV. We explored attitudes to and experiences of tracking and sharing PHD in order to identify the challenges and opportunities within HIV care in the United Kingdom.

**Methods:**

We conducted a qualitative study comprising 24 semi-structured interviews with service users (SUs) (n = 10) and healthcare professionals (HCPs) (n = 14) between February and November 2020. Transcripts were analysed collaboratively using Thematic Analysis.

**Results:**

There was wide variation in the extent and types of PHD tracked and shared, and how this was done. Key themes included the use of PHD to enhance empowerment and self-knowledge about health, PHD enabling better clinical care, PHD impacting clinical consultations and SU-HCP relationships, the burden of PHD tracking, and privacy and data security concerns.

**Conclusions:**

Our findings highlight the opportunities and challenges of tracking and sharing PHD in the context of HIV, especially in view of increasing remote and digital clinical care throughout the National Health Service. Opportunities included enhanced autonomy and control over health and facilitating improved relationships and communication between SUs and HCPs. However, these opportunities must be considered in the context of constraints of service delivery and potential burden to SUs and HCPs, as well as key challenges regarding privacy.

## Introduction

In 2023 there were an estimated 39.9 million people worldwide living with Human Immunodeficiency Virus (HIV) and 3.1 million people living with HIV in Europe.^1^ In the same year in England, approximately 100,000 people were living with diagnosed HIV infection and accessing care; 98% were virally suppressed (also known as having an ‘undetectable’ HIV viral load) on HIV treatment (antiretroviral therapy (ART)) meaning that they will have a normal life expectancy, and cannot pass on HIV to sexual partners.^2^ HIV is therefore no longer universally life-limiting and is instead a long-term condition (LTC) where people have access and adhere to ART.^3^ However, this requires high levels of adherence to lifelong medication and regular engagement with clinical services. Further, over half of people receiving HIV care in England in 2023 were aged 50 years or over.^2^ As people living with HIV reach older age, they increasingly experience age-related comorbid conditions including cardiovascular disease, renal disease, osteoporosis and neurocognitive problems, meaning that ongoing care and monitoring becomes even more important.^4–6^

In the United Kingdom, specialist HIV care is provided free of charge by the National Health Service (NHS) to all people diagnosed with HIV, regardless of migration status. The long-term management of people with stable HIV is typically supported by a 20 to 30 minute consultation with a specialist HIV healthcare professional (HCP) twice a year; some of these appointments may be remote (by phone, email, text and/or video). These consultations are informed by results from blood tests prior to the appointment, weight and blood pressure readings taken in the clinic, and information reported by service users (SUs) such as symptoms and medication adherence, often communicated verbally during the consultation.^7^ The need for long-term engagement in clinical care and monitoring, coupled with fewer and briefer HCP appointments and more remote care, mean that tools to record, track, and share personal health data (PHD) may offer opportunities for people living with HIV to optimise their health and wellbeing.

The collection and sharing of PHD by individuals is increasingly commonplace. We define PHD as information about physical and mental health that may be self-recorded by individuals^8^ or HCP-collected as part of the personal health record.^9^ Health and wellbeing related information including step counts, heart rate, sleep and menstrual cycle are routinely captured by many people. This is not a new phenomenon, with people long recording such data in writing as notes or in diaries. However, the proliferation of digital technologies including mobile phone applications (apps) and wearable devices such as smart watches has facilitated and broadened the collection of PHD, making them an ever more pervasive part of our social lives.

Collecting PHD can be particularly useful for people living with LTCs such as HIV, who may wish, or be encouraged by HCPs, to monitor daily behaviours, symptoms, medication use, side effects and responses to treatment. Such tracking has the potential to offer a number of opportunities for improving the self-management of LTCs and, when shared with HCPs, can improve clinical care.^10,11^ Individuals may also keep records of *HCP-collected* data, such as blood test results, radiology reports and blood pressure readings taken within the clinic, for their own personal use and to facilitate sharing between often decentralised health systems. In the United Kingdom, for example, such data is increasingly accessible in real time via the National Health Service (NHS) app^12^ or other online personal health record portals.^13,14^

The current turn towards PHD tracking has roots in the Quantified Self Movement of the late 2000s.^15^ Quantified Self, and other related movements, sought to optimise health and wellbeing through the tracking of the body, motivated by the premise that knowledge of these data would allow individuals greater control over their health. This emphasis on data tracking for the self-monitoring of health is particularly pertinent at the present time, as people living with LTCs have fewer opportunities and less time to spend with their HCPs. There is consequently increased focus on self-management of LTCs outside clinical spaces.^16^ A key focus of the UK's national strategy for healthcare is empowering self-management amongst those with LTCs through the use of digital technology and remote monitoring.^16^ Digitally enabled remote monitoring and management has further accelerated in response to the COVID-19 pandemic and the consequent, and enduring, changes to how clinical care is delivered.^17^

The changes in health service delivery in the United Kingdom in response to the 2019 NHS Long Term Plan^16^ and accelerated by rise of remote care during the COVID-19 pandemic, lend an urgency to the development of an evidence base on PHD tracking and sharing amongst people with LTCs. This is particularly true for LTCs that disproportionately impact marginalised populations, who may also experience digital inequalities.^18^ As shared responsibility and self-management are key priorities in the Long Term Plan, alongside new options for accessing care digitally, it is important to understand the opportunities, challenges and unintended consequences of such PHD data tracking and sharing amongst people with LTCs in the United Kingdom. This is all the more so as these shifts have been underscored by the recent 10 Year Health Plan for England, which highlights the role of the NHS app in facilitating service user access to their health data as well as giving renewed focus to the potential of wearable devices and mobile apps to monitor health.^19^

There is limited data on the challenges and opportunities afforded by the tracking and sharing of PHD in HIV specifically, with most of the available literature focussing on mobile health (mHealth) platforms or electronic personal health record portals,^20–25^ many of which combine access to health data with the ability to directly contact HCPs. This literature highlights the opportunities of PHD to support better understanding and management of ones’ own health,^22,23^ including through monitoring health behaviours and laboratory results^21^ and has demonstrated the value of these tools in supporting improved health outcomes.^24,25^ Elsewhere, PHD tracking and sharing in HIV care has been found to facilitate and support ‘whole-person’ care.^26,27^ However, when exploring the potential for PHD to improve HIV care, it is important to consider the role of HIV-related stigma. Despite advances in treatment, life expectancy and quality of life, HIV remains a stigmatised condition. This stigma is amplified by the multiple intersecting disadvantages that may be experienced by many people living with HIV as a result of their gender, ethnicity, sexuality, drug use, sex work and/or immigration status.^28^ One of the key challenges in terms of the tracking and sharing of PHD by people living with HIV is that stigma may pose a significant barrier, due to concerns about privacy and security, which are often related to fears about HIV-related stigma.^23,29–34^ On the other hand, technologies such as personal health record portals may remove the necessity for postal or telephone contact from HCPs, which may facilitate increased privacy where there are concerns family members might intercept messages by post.^20^

In this article, we describe perspectives on and experiences of tracking and sharing PHD among people living with HIV engaged in NHS clinical care and their HCPs. We explore the perceived challenges and opportunities presented by the use of PHD within HIV care and consider their implications for the rapidly shifting delivery of HIV clinical care within an increasingly digital health service.

## Methods

This qualitative interview study is part of a broader programme of work from *INTUIT: Interaction Design for Trusted Sharing of Personal Health Data to Live Well with HIV,*^35^ which was funded by the Engineering and Physical Sciences Research Council (EPSRC, ref: EP/R033900/2). The aim of INTUIT was to understand the needs, experiences and concerns of people living with HIV and other long-term conditions, and their HCPs, to design supportive tools for the collection, tracking and sharing of PHD, with a particular focus on trust, identity, privacy and security (TIPS).

### Study design and inclusion criteria

In this article we present data from 24 semi-structured interviews (SSIs) with service users (SUs) (n = 10) and HCPs (n = 14). These took place between February and November 2020, notably stretching from the period before, during and after the first COVID-19 lockdown in the United Kingdom, which began on the 23 March 2020.

The study protocol was reviewed and approved by the Health Research Authority (HRA) (ref: IRAS 271133 19/YH/0417) prior to study commencement and participant recruitment. Ethical considerations included the preparation of documents to support obtaining informed consent from individuals to take part, understanding what this would involve, and agreeing to how data collected from participants would be managed, stored and used for the purposes of this research. In response to COVID-19 lockdown measures and related implications for assessing risk, the study team revised methods (including to conduct interviews online), obtaining additional HRA approval for making these amendments led by Chief Investigator (AD) and Principal Investigator (JG).

The SUs were eligible to participate if they were aged ≥18 years, living with diagnosed HIV infection, attending HIV services at the recruiting clinic, and able to complete the consent process and study activities in English. Individuals were ineligible to participate if they were under 18 years of age, were not living with diagnosed HIV infection, were not attending HIV services at the recruiting clinic and were not able to complete the consent process and study activities in English. Participants were not known to the interviewer before being approached to take part in the study. Given that recruitment was ongoing during the first COVID-19 lockdown, convenience sampling was used for SU interviews, meaning that participants were recruited if they were happy to participate, regardless of their demographic details.^36^ Interviews explored beliefs about and experiences of capturing, using and sharing PHD through both digital and non-digital means (see Supplemental material).

HCPs were eligible if they were HCPs (e.g. consultant, non-consultant grade doctor, nurse, pharmacist, psychologist, social worker or health adviser) providing HIV outpatient services within the recruiting clinic. It was assumed that all eligible HCPs would be able to participate in an interview in English and were able to give their consent to participate in the research. Individuals were ineligible to participate if they were not an HCP providing HIV care at the recruiting clinic. Notably, the recruiting clinic is part of the NHS, meaning all participating HCPs provided care within the publicly funded and freely available healthcare system. Participants were not known to the interviewer before being approached to take part in the study.

Sampling for HCP interviews was purposive, meaning that we selected participants to invite to interview in order to ensure we had representation from the breadth of the multidisciplinary team in terms of staff role and gender.^36^ Interviews explored beliefs about and experiences of SUs capturing and sharing PHD with them in the course of the care they provide, through both digital and non-digital means (see Supplemental material).

### Ethical considerations

Prior to participating in the study, all participants were provided an information sheet which outlined the study aims and how their data would be processed and managed. All participants were given the opportunity to ask any questions and were reminded of the voluntary nature of their participation, emphasising that their care would not be affected in any way should they decide not to participate. Prior to participating, all participants either signed a paper version of the informed consent form (if in person) or in the case of remote interviews, gave their consent verbally, meaning that the researcher read each question on the consent form out loud and the participant affirmed that they were in agreement to all aspects of the study. The latter method was audio recorded and stored separately to interview audio and transcripts. Interview audio recordings were transcribed by a professional transcription company, who had signed a confidentiality agreement, before being checked for accuracy by a member of the research team.

### Participant recruitment

Participants were recruited from one London HIV clinic. SU participants were identified from a list of SUs who had previously consented to being contacted about participating in research. KCL contacted all eligible participants to explain the purpose of the research and what would be involved in taking part; those who agreed to participate were scheduled for an interview. SU participants were offered a £20 shopping voucher in recognition of their time.

Interviews with SUs (n = 10) took place both face-to-face (n = 2) and remotely by phone or video conference (n = 8). Both face-to-face interviews took place prior to the first UK COVID lockdown in March 2020; remote interviews took place in autumn 2020 after the first lockdown. Interviews lasted between 38 and 86 minutes, with the average length of interview being 52 minutes.

Interviews with HCPs (n = 14) took place both face-to-face (n = 9) and by phone or video conference (n = 5). Those that took place face-to-face occurred prior to March 2020, while those that occurred remotely took place either during or after the first UK COVID lockdown. Interviews lasted between 29 and 113 minutes, with the average length of interview being 54 minutes.

### Data collection

Face-to-face interviews were conducted in the research team's offices. When interviews took place remotely, they were conducted either by phone or video conferencing (via Zoom), according to participant preference. All interviews were conducted by KCL.

All interview participants completed a short online demographic questionnaire prior to beginning the interview, which was administered using Research Electronic Data Capture (REDCap), a secure web application for developing and managing online surveys. Qualitative data collection continued until saturation had been reached.

### Data analysis

We analysed demographic data from both SUs and HCPs descriptively using REDCap and Microsoft Excel. We analysed interview transcripts thematically^37,38^ using NVivo 12 qualitative data analysis software as well as written summaries. Prior to coding, we all read each transcript in order to achieve data familiarisation, allowing all researchers to become ‘intimately familiar with the data’.^39^ The initial coding phase involved applying short descriptive labels (‘codes’) to segments of the text in order to identify where topics were repeated. A proportion of transcripts were coded manually in Microsoft Word to allow cross-checking within the team before developing a codebook for each set of interviews in Nvivo. We then applied initial deductive and inductive codes to the data in Nvivo. Deductive codes, meaning codes which we had developed *a priori* to analysis, related to specific PHD types of interest (e.g. discussions of sharing data related to weight or menstrual cycles) and issues of concern identified within the literature (e.g. privacy and security issues). Inductive codes were developed through engagement with the interview transcripts and through discussion amongst authors. The authors applied inductive codes primarily to sections of text related to experiences of and beliefs about PHD capture and sharing. EJN (cis female Research Fellow, Medical Sociologist, PhD), KCL (cis female Senior Research Fellow, Medical Sociologist, PhD), KH (cis female medical student) and AM (cis male medical student) analysed SU interviews, and KCL analysed HCP interviews.

In addition to formal analysis using Nvivo software, researchers also wrote analytic summaries of interviews while reading, and with subsequent rounds of coding and writing up summaries of key themes, the team developed the key categories ‘opportunities’ and ‘challenges’. EJN and KCL synthesised findings across both sets of interviews using analytic summaries and coded NVivo files to integrate findings from both SU and HCP perspectives. Analysis was guided and supervised by ST (cis female Senior Clinical Research Fellow, medical anthropologist and consultant physician in HIV) and JG (cis female Senior Clinical Research Fellow and consultant physician in sexual health and HIV).

All authors discussed interview data in-depth within team meetings until consensus was reached on the key themes around PHD self-capture, use and sharing as described by both SU and HCPs. Emphasis was placed on ‘following the thread’ between the two datasets. For example, where SUs identified a key challenge of PHD, this theme was sought out in the HCP dataset and compared. Points of congruence and conflict across the datasets underpin the key findings presented here.

All demographic questionnaire responses, interview recordings and transcripts were pseudonymised using a unique participant identification number that can only be linked back to the identity of the participant via a participant key. This key is stored separately in the university Data Safe Haven, which is certified to the ISO27001 information security standard and conforms to NHS Digital's Information Governance Toolkit, as well as behind the NHS firewall at the clinic site.

Prior to analysis, interview transcripts were pseudonymised by removing all potentially identifying information (e.g. name, location and occupation title).

## Results

### Sample characteristics

SUs were mainly aged 45 and 64 years, of White ethnicity and UK born (Table 1). A high proportion of SU interviewees were men who have sex with men (n = 7), reflecting the epidemiology of HIV in the United Kingdom and in this clinic setting. All were taking ART and had an undetectable viral load, indicating that they were adherent to their medication. HCP participants included HIV doctors, health advisors, specialist nurses, a specialist HIV pharmacist and a clinical psychologist. Four HCP participants were women, 3 were men and the time they had worked in HIV care ranged between 3 and 19 years. HCPs worked across a range of settings within the HIV outpatient clinic, including in specialist clinics, such as those for young people and those with co-morbid conditions (e.g. viral hepatitis). Age and ethnicity were not collected for HCP participants as it was felt that this was likely to make them identifiable, and exact ages of SU participants were not collected in order to keep identifiable information to a minimum.

**Table 1.** Sample characteristics.

Sample characteristics	Service users (n = 10)	Healthcare professionals (n = 14)
Age (years)		
18–44	1	-
45–64	7	-
≥65	2	-
Gender (self-reported)		
Female (inc. trans)	2	8
Male (inc. trans)	8	5
Prefer to self-describe^a^	0	1
Men who have sex with men	7	-
Ethnicity (self-reported)		
White UK/white other	8	-
Black African	1	-
Other	1	-
UK-born	7	-
On antiretroviral therapy	10	-
Undetectable HIV viral load (self-reported)	9	-
Duration of HIV work (mean years)	-	12
Clinical role		
Consultant HIV physician^b^	-	5
Non-consultant grade HIV physician^c^	-	3
Specialist nurse	-	2
Health advisor^d^	-	2
Pharmacist	-	1
Clinical psychologist	-	1

^a^Self-described as intersex.

^b^ Senior doctor who has completed clinical training who oversees medical students and other doctors in training.

^c^ Qualified doctor who has obtained a medical degree and is in the process of completing specialist clinical training.

^d^ Health advisors most often have training in nursing, social work or counselling who provide follow-up information and psychosocial care for people living with HIV to complement clinical care.

### Data tracking and sharing practices

The extent to which SUs tracked their PHD and the types of data they recorded varied considerably; some reported tracking a range of PHD, whereas others recorded very little or none. Several SUs kept detailed records of PHD such as weight, mood, blood pressure, sleep and/or perceived medication side-effects like diarrhoea. Some SU participants used home blood pressure monitors to keep track of their blood pressure between clinic visits, often in addition to taking their blood pressure on the day of their appointment using the clinic machine. These data were recorded in a range of formats including notebooks, diaries, electronic spreadsheets, and in the notes feature of smartphones. Several SUs reported using wearable fitness trackers and/or health apps on their phone to track activity, heart rate, sleeping pattern, steps per day and/or food intake. Reflecting the time frame of data collection (February–November 2020), a few participants also reported formally recording (or mentally noting) body temperature and oxygen saturation levels due to concerns about COVID-19.

HCPs recognised that SUs were likely to be using apps to track PHD, but most commented that these were rarely shared and discussed in clinical consultations. A notable exception was menstrual tracking data, which HCPs stated were the most common app-derived data to be shared during consultations, largely as an aid to remember the date of last menstrual period. However, no SU interviewed reported using menstrual tracking apps, which is consistent with the gender and age profile of participants in this study. Some HCPs were also aware of patients using apps to support behaviour change, for example, alcohol reduction or smoking cessation, but again stated that such data were not routinely shared and discussed during consultations.

As well as the PHD described above, SUs reported recording and sharing information about potentially HIV-related symptoms (such as fatigue), medication side-effects and medications they were taking in addition to antiretrovirals. HCPs perceived medication history as the most useful data routinely shared; it needs to be updated within the patient record during each consultation to ensure there are no potential interactions between HIV and non-HIV medications. Medication history was shared in a variety of forms including verbally from memory, in photographs, by referring to handwritten or typed lists or by bringing medications themselves to the consultation. However, the recording and sharing of medication data was not consistent:

'*Some people do [record their medications]. Some people are quite good at it. And some people don’t. It is quite variable. And you do get a paper copy once you do hand in a prescription. So they have a list of their repeated medications, which they sometimes carry in their purse or whatever’. (HIV pharmacist)*

HCPs noted a wide variation in the degree of tracking and sharing of these kinds of PHD amongst SUs, with some recording their PHD meticulously:

'*Some people are quite amazing. Some people have a booklet of the medications they are taking from us [and] the medications they are provided by their GP. And the blood results of every six months. So basically they have a booklet - I saw this guy last week, so we started talking and then I said, “Okay, your viral load is undetectable.” And he was like, “I’m undetectable. You see before it said I had 150.” So he just wrote down every single blood result. And it was a booklet … That's something that I have seen a few times’. (HIV specialist nurse)*

However, this level of detailed data capture was not the norm. Amongst SUs who did not formally or comprehensively track their PHD, some described making a mental note of how they were feeling, whether their weight had fluctuated, and/or took ad hoc notes about mood or side effects in diaries. Of those who reported not routinely collecting PHD, some had previously done so, but had stopped as they felt it wasn’t utilised by HCPs. Others felt that these data were no longer relevant as they had no current health concerns, sometimes reflecting an ‘acceptance’ of living with HIV:

*‘I used to write things down actually. But I must say I’ve sort of stopped now. Because, you know. I suppose I’ve accepted everything. I’ve learnt to live with it [HIV]. And so long as I tick along the same, then I’m fine really.’ (Service user, 65+ years old gay White man, living with HIV for 13 years)*

HCP-collected data, such as blood test results, were valued by most SUs. They described HCPs sharing their results on a computer screen while discussing the results with them. A small number of SUs asked their HCP to print blood results, so they could save it in their own records and/or share it in hardcopy form with other HCPs, such as in primary care or another specialty; others brought a notebook to appointments to write down their results. One SU reported that they revisited these records, comparing their results over time and sometimes also looking online to find out more about any results that were outside the normal range.

### Enhancing self-knowledge and autonomy and enabling better care

Experiences of engaging with and the value attributed to self-collected PHD were diverse across both SU and HCP participants. Self-tracking and sharing of PHD was perceived as having the potential for SUs to increase self-knowledge about health, wellbeing and their bodies, to enhance ownership over data, and to develop further autonomy in managing HIV and other LTCs, as well as improving service delivery. Some SUs tracked PHD in response to a particular health concern, for example, potential medication side effects, so they could later share these data during a consultation. In this sense, PHD tracking not only facilitated an individual's greater understanding of their own health and wellbeing, but had the potential to enhance their ability to self-manage. For example, some SUs commented that PHD tracking generated insights into the effects of their antiretrovirals, allowing them to identify and manage potential side effects.

Weight gain in relation to ART was a particular concern for SUs, which is pertinent given the increasing prevalence of obesity amongst people living with HIV, which is partly attributable to some antiretrovirals.^40^ One SU described how calorie tracking had enabled them to identify their ART as a likely cause of their weight gain rather than diet. Similarly, another SU, aware that weight gain was a potential side effect of their new medication, made the decision to switch antiretrovirals after tracking their weight over time:

*‘I realised that the weight gain was just unsustainable. It really, it helped me to, it helped my decision in terms of just you know not continuing on that treatment.’ (Service user, 55–64 years old heterosexual Black woman, living with HIV for 32 years)*

However, for many SUs who tracked their PHD, capturing and reflecting upon that data was more a personal act of self-knowledge than one that initiated particular decisions about their clinical care. The vast majority of SUs who tracked PHD, collecting information such as sleep, weight, diet and step counts, described doing so to better understand their bodies, and to obtain objective confirmation of their embodied subjective experiences.

*‘… I was having such bad sleep … if I’m going through a particularly bad few nights sleep, then I’ll put it on just to see if it can tell me anything … I sort of listen to it back and think, oh yeah, I didn’t sleep very well.’ (Service user, 45–54 years old gay White man, living with HIV for 16 years)*

HCPs echoed this perception of PHD tracking as a tool for developing self-knowledge, highlighting this could lead to greater autonomy and motivation around health and wellbeing, and enhancing empowerment through a sense of ownership over their health and their data. Several HCPs discussed how SUs could gain a better understanding of appropriate healthy targets through PHD tracking, providing impetus to address any concerns:

*‘I think it's good because it's showing that they’re showing ownership of their condition as well and they’re being proactive. And you know, it's all part of their education in terms of saying, what should a normal blood pressure be? So, it's just, it feels like they’re getting more involved in their health as well. And they know what to look out for, so they know what normal is’. (HIV consultant doctor)*

Some HCPs emphasised how the value of PHD self-tracking lay in its potential to enhance self-management of health outside of the consultation, in which time is very limited:

*‘You’re only seeing someone for 40 minutes a year, that's not very long, in their whole lives, you’ve got a tiny amount, a little tiny mini bit of their lives that you’re involved in … so you’ve got to think of things that sort of enhance that, and things that you can leave with them to take care [of]’. (HIV consultant doctor)*

Many participants, both SUs and HCPs, felt that this enhanced understanding and ownership over SUs bodies and health had the potential to enable better clinical care. In this sense, more information meant better and more personalised care, as this SU described:

*‘The more information they have about me, the more they’re going to be able to help me. So if I keep things hidden, or if I don’t disclose whatever, there may be something that doesn’t seem to be important to me that is in fact important to them.’ (Service user, 55–64 years old heterosexual White woman, living with HIV for 21 years)*

Many SU and HCP participants described that PHD tracking and sharing within consultations facilitated better care by enhancing recall of experiences that may have occurred weeks or months prior, thus improving the accuracy of information and experience relayed in twice yearly consultations, and also generally improving communication between HCPs and SUs.

*‘I think it improves the communication between the clinician and the patient, and the understanding of what was discussed, because yeah you’re sort of more on the same page when you’re looking at data that they’ve brought in and putting it onto the system’ (non-consultant grade HIV doctor)*

### Impact of personal health data on the clinical consultation

Despite the perceived value of making space in the consultation for discussions of self-tracked PHD as acknowledged above, many HCPs described the practical challenges posed by the additional workload created by reviewing and triaging self-tracked PHD that SUs shared with them. Sifting through and interpreting large volumes of PHD requires time and cognitive effort, in consultations that are already significantly constrained by time:

*‘When someone comes with a diary with everything that's gone on, and they go through that and that becomes very difficult; you end up not seeing the wood for the trees, it becomes too dense and complicated’. (HIV consultant doctor)*

While managing the workload created by the sharing of PHD posed challenges within the consultation, some HCPs also highlighted that such an interaction could help them to identify when a SU needed more care and support. When asked how such a situation might be handled in the context of care delivery, the HIV consultant quoted above described that a SU bringing a large amount of PHD to a consultation, whether relevant to their HIV care or not, may flag up heightened needs either for further investigation or more engagement and support, to address these concerns:

*‘We have different ways of managing that and seeing people more frequently and stuff, because clearly that's some kind of manifestation of problems’. (HIV consultant doctor)*

Summarising both the potentially positive as well as the negative impacts of sharing PHD on clinical consultations in HIV care, one HCP emphasised the role of the clinician in considering the instrumental and relational value of PHD along with the practical impacts on workload and service delivery.

*‘It can be exactly on two sides of the coin, it can certainly make things a lot more efficient and easy to document, but then yes it can be the opening a can of worms effect, but yeah, but then that's the skill of the clinician to kind of contain what's relevant to that specific appointment’. (non-consultant grade HIV doctor)*

Another HCP addressed the complexities of managing information within consultations in relation to SU PHD. They described an instance when reviewing a large volume of PHD was not directly relevant to HIV care and took up a great deal of time in the consultation. However, these PHD ultimately allowed the HCP to re-direct the SU to seek care from their general practitioner (GP) for non-HIV related concerns.

*‘On one hand, it can help with the time constraints of a consultation because you can reprioritise or see what needs to be addressed, and occasionally it can backfire and actually bring in so much and there's so much going on that it can complicate [care in the consultation], so by the time you’ve looked through and then decided, “well actually although that's important to you, it's not really an HIV issue”, so obviously you can get a bit bogged down in things that aren’t always relevant’. (non-consultant grade HIV doctor)*

Several HCPs noted that historically the HIV clinic has acted as a ‘one stop shop’, addressing a broader set of clinical and social care needs for people living with HIV that might otherwise have been routinely addressed by other HCPs or organisations. They were therefore accustomed to discussing non-HIV related health concerns in consultations; many perceived this central to the delivery of high quality holistic HIV care as well as part of the work of triaging to primary care and other specialties.

### Impact of personal health data on the clinical relationship

Both HCPs and SUs recognised that sharing PHD in HIV care was more than a transactional exchange of information; rather, it was an important part of, and had the potential for significant effects on, the SU–HCP relationship. Beyond the relational benefits of assuring that SUs were heard via the sharing of their PHD in consultations, HCPs described the sharing of information that might initially appear irrelevant or tangential to HIV care, for example, about family life, relationships, day-to-day living or important life events, as valuable for building rapport within an ongoing HCP–SU relationship that may often extend over several years.

Nearly all SUs who reported that they previously shared their self-recorded PHD with their HCPs but no longer did so, stated that was because they did not feel their HCP devoted sufficient time and attention to their PHD and did not act on concerns they raised when sharing.

*‘Because basically I had nowhere to give this information. Nowhere to use it. And when I did show it sometimes, they just looked at it and said, “Yeah that's like fine.” Or you know, “Not that interested.” So I didn’t bother anymore’. (Service user, 65+ years old gay White man, living with HIV for 13 years)*

This potential for frustration experienced by SUs was acknowledged by a number of HCPs, who discussed how they sought to manage it in a way that would minimise negative repercussions on the SU–HCP relationship. One HCP described a situation where a SU shared a large amount of data on a health condition which the HCP felt was not relevant to HIV care:

*‘I kind of thanked him for bringing it. And we went through some of the summarised printed sections, like the graphs over time. And I kind of let him know that I wasn’t going to be able to read every single prose diary entry, but we glanced through a few together and talked about the overall trends. And I think he had [been] a bit cross that at appointments with other health professionals, they had not wanted to look at his recordings. So it was important to him, definitely, that it was reviewed’. (non-consultant grade HIV doctor)*

Another HCP described similarly how it was important in consultations to let SUs be ‘heard’ with regard to sharing their data, even where it may not be relevant to the care they are providing. Here, the HCP emphasised the value of taking time for PHD sharing in delivering care that is ‘holistic’ and enhances comfort and rapport.

'*Sometimes they just want to tell you what they want you to know, which as you said, may not be relevant. But that's, again, healthy. I don’t mind. I think we should be holistic. So if that way it made him feel comfortable, wonderful, tell me’. (HIV specialist nurse)*

A number of HCPs described circumstances when SUs shared their PHD when they as HCPs did not personally feel it was relevant to the care they received in the HIV clinic, but they made space within the consultation for this exchange because it was an important way of acknowledging the SU's concerns and needs.

### Perceived burden on service users

Although time was primarily a concern of HCPs, SUs also perceived PHD tracking as potentially time-consuming. As a result, they felt they would struggle to engage unless there was a clear need or agreed purpose:

*‘Whether I’d find it very time-consuming, and whether I could actually be bothered to do it, if you know what I mean. I’m quite happy doing the step count because it takes a minimum amount of effort. But to sit there and weigh yourself and stuff like that, I mean it's, you know I’d find- I don’t know if I’d be regimented to do it’. (Service user, 55–64 years old heterosexual White man, living with HIV for 26 years)*

HCPs also reported that some SUs did not collect certain types of data that they had recommended tracking, such as sleep or diet:

*‘If you’ve asked them, have you noticed this trigger, that trigger, this trigger, that trigger? And they’re saying no they haven’t really noticed that. Or I haven’t really observed it. I’ll say to them to sort of try and hone into and try and observe when you get it. Are there circumstances around it? Keep a diary. No patient, I think, has ever brought back a diary of it’. (HIV consultant doctor)*

One HCP described asking SUs to keep track of their PHD between consultations as ‘like giving them homework to do’, emphasising the anticipated burden of time and effort. Another HCP reflected that ‘the days of diaries [is] now kind of dying’, also noting the inherent tensions in asking SUs to keep diaries about issues such as adherence to medication.

*‘I think the irony of it is, is that if someone is not compliant, they’re probably not going to get a diary and start writing about their compliance’. (HIV pharmacist)*

### Privacy and data security concerns

There was an awareness both amongst SUs and HCPs of the implications of PHD in their lives beyond the clinical space. While some SUs were publicly quite open in their personal lives about living with HIV, others expressed concerns about keeping PHD records, both digitally and on paper, that contained data that let others know that they were living with HIV. This also included using applications that hinted at an HIV-related purpose in its design.

For some, this was because they had not yet shared their HIV status with people close to them who would be most likely to have access to their devices. One participant, a man who had not told his wife of over 20 years about his HIV status, spoke about his discomfort at recording HIV-related PHD or installing an HIV-specific app on his phone:

*‘I’d feel very uncomfortable. I couldn’t leave my phone around. I mean, we both know each other's passwords into our phones. I certainly wouldn’t want my wife or somebody to pick up my phone and say, can I just have a look at that? And I certainly wouldn’t want that to be one of the apps on my phone. I mean it's totally for that reason. And also my [younger family members], they do quite often get hold of my phone and will sort be on my phone. So no. No definitely not … I’ve kept this very much to a very select amount of people. I certainly wouldn’t want it on my phone, no’. (Service user, 55–64 years old, heterosexual White man, living with HIV for 26 years)*

HCPs broadly echoed these concerns about privacy and security, with one HCP highlighting that if SUs were recording HIV-specific data on a digital device, these SUs might wish to apply extra security, like a password or fingerprint recognition, to protect access to these data.

Yet for others, there was an acceptance of the ubiquity of personal data sharing of various sorts in modern life. They conceived of PHD, even data related to HIV, as similar to the multitude of data types they already routinely shared and did not express any particular concerns about tracking and sharing them, even in this case, with third party commercial entities, if there was a perceived personal benefit:

*‘I don’t care to be honest. I’ve got nothing to hide. And if something can help me whether I know I need the help or not, what's the issue?’ (Service user, 45–54 years old, gay White man, living with HIV for 16 years)*

## Discussion

In this article, we present results from a qualitative study exploring the challenges and opportunities of PHD for specialist clinical HIV care and the experiences and perspectives of SUs and HCPs. We found that while people living with HIV report tracking and sharing of data such as medication side effects and weight gain, this practice was highly variable in terms of type of data recorded and shared as well as quantity and frequency. When data were shared in the consultation, it was often those data types that are routinely elicited as part of routine clinical care, for example, blood pressure, weight, concomitant medication and menstrual cycle. The sharing of blood test results by HCPs to SUs was particularly valued by SUs, potentially reflecting the longstanding clinical and symbolic significance of CD4 count and HIV viral load as markers of immunological recovery and viral suppression, respectively, indicating treatment success.^41^

A key opportunity offered by the tracking of PHD, recognised by both SUs and HCPs, was the potential positive impact on health and wellbeing, as it was seen to foster enhanced autonomy and control over one's health. It was perceived to motivate people to look after their health, as well as being a tool to deepen understanding of ones’ body. These findings echo those on broader discourses of PHD self-tracking that focus on embodied knowledge of the self to enhance health and wellbeing, in particular the Quantified Self movement with its emphasis on a citizen science of ‘self-knowledge through numbers’,^42^ as well as other work focussed specifically on HIV.^27^ PHD sharing within consultations was also conceived as potentially improving clinical care, allowing it to be more holistic and better integrated with both primary care and other forms of specialty care. This finding may be due to the well documented relationship between PHD self-tracking and improved engagement in other clinical contexts^43^ as well as facilitating more user-centred, personalised care.^44,45^

Beyond these instrumental benefits, PHD sharing in routine HIV clinical care offers opportunities for developing and consolidating SU–HCP relationships. HIV care may be an especially powerful site for research on the value of relationship building and rapport around PHD sharing because of the historically stigmatised nature of HIV^28^ and the nature of HIV care, which involves regular interaction with a team of health professionals, potentially from early adulthood and throughout the life course.^7^ Our data suggest that PHD sharing in HIV care might be seen as a powerful way to build trust and rapport, validating people's lived experiences of a long-term condition. This was evident in the way that our HCP participants described making space for the sharing of PHD, even when it was not considered medically relevant to HIV care, as well as the frustration SU interviewees felt when the PHD they had collected was not taken into account. On a more tangible level, the shared work between HCP and SU of making space for and interpreting PHD within the consultation can itself be an important means of receiving emotional support and recognition.^46^ This reflects the findings of other analyses conducted as part of our study, which make clear the impact of trust and relationship on whether or not data is shared, as well as the potential difficulties of developing these trusting relationships in the context of remote care.^26,27^

Our findings highlight critical challenges to the tracking and sharing of PHD by people living with HIV. Notably, SUs commonly opted out of collecting data that their HCPs had advised them to track, perceived by some HCPs as being a result of the burden of the request. Furthermore, some initiated self-tracking to better understand an aspect of their health but stopped when they felt their HCPs did not engage sufficiently with the sharing of these data. These findings echo those on PHD self-tracking amongst people with other LTCs, where collecting PHD often feels like ‘work’ for SUs, meaning it is unsurprising that it is a practice that can be a hard to sustain over time.^47^ They also highlight how the opportunities for improved care facilitated by PHD are only possible where this can be supported and maintained and may indicate limited opportunities for reflection afforded in current tools.^48^

The sharing of PHD, particularly when HCPs deemed it irrelevant to HIV care, placed additional time and cognitive burden on HCPs, at a time when consultations are already constrained. This also reflects findings of a scoping review on patient generated PHD in HIV, which highlighted that although HCP engagement with this data tended to be welcomed by SUs, HCPs were often concerned about the negative impact on workload.^49^ SUs in our study expressed frustration and disappointment when they perceived that HCPs had ignored PHD they had shared in consultations. HCPs in our study were aware of the potential for this to undermine the SU–HCP relationship, deterring the SU from tracking PHD in the future. This highlights how PHD sharing may be an important practice around which to negotiate the SU–HCP relationship, emphasising the value in SU and HCPs priorities being aligned,^50,51^ and indicating the importance of teaching HCPs how to manage sharing of such data. As the recent NHS 10 Year Health Plan for England promises to increasingly incorporate digital tools including wearables, smart devices and mobile apps into the healthcare system, it is likely that these skills will become increasingly relevant and necessary.^19^

Because of the historically stigmatised nature of HIV,^52^ self-tracking and sharing of PHD that is identifiably HIV-related including measures that are uniquely associated with HIV, for example, CD4 count or HIV viral load, presents important privacy and security challenges.^26,27^ These concerns were largely related to SUs fear of others finding out about their HIV status, particularly where they had not shared their status with people close to them.^31^ Worries about privacy and security, however, were not universal amongst our participants and there was a recognition of both the ubiquity and utility of data sharing in modern life.^22^ Other work on data sharing in the context of health have used the privacy calculus model, which posits that users will be more likely to take up a technology if the benefits of sharing information outweigh the costs to privacy, with some extending this model to include general attitudes towards privacy.^53^ Some authors have found there may be a higher perception of the benefits for chronic than acute conditions, and that here trust in provider is also more likely to influence this calculus.^54^ Our data highlight the relational context of both the opportunities and challenges of PHD as they are navigated in practice by both SUs and HCPs, underscoring the importance of trust and relationship in navigating these concerns.

### Limitations and strengths

This study was significantly impacted by the changes to service delivery as a result of COVID-19 lockdown measures, resulting in fewer participants than had been planned. However, importantly, data collection continued until saturation had been reached. Most of our participants were men who have sex with men, reflecting the epidemiology of HIV in the United Kingdom. However, our analysis would have benefitted from the inclusion of more women and people from racially minoritised communities. Finally, we recruited participants from the service user population of one HIV outpatient clinic in London. This study, therefore, may not reflect the experiences of those outside of London and does not capture the experiences and needs of people living with HIV who are not engaged in care.

While the COVID-19 pandemic presented recruitment challenges and required amendments to our procedure, our study uniquely spanned the first nine months of the pandemic and captured valuable perspectives on and practices of PHD self-tracking and sharing at a time when HIV care, and healthcare in the United Kingdom more broadly, had swiftly pivoted to remote service delivery. By synthesising both SU and HCP perspectives, we are able to give a more holistic account of the potential challenges and opportunities of PHD tracking and sharing in HIV clinical care.

## Conclusion

PHD has long been collected and shared between multiple actors within and beyond the HIV outpatient clinic, including public health surveillance and other healthcare providers such as General Practitioners. Our analysis highlights what people living with HIV and their specialist HCPs considered to be the opportunities of collecting, tracking and sharing of PHD in consultations. The sharing of PHD between SU and HCP may enhance knowledge of one's own body, improve engagement in care, facilitate more efficient care, and enhance SU-HCP relationships.

However, we also identified several critical challenges. PHD sharing in consultations comes at a cost in terms of time and cognitive burden on both SU and HCP. Therefore, the use of PHD must be considered within the wider context of increasing pressures within health services. Although HCPs were generally positive about their ability to navigate the extra work that such practices might open up, this could pose a significant challenge should the quantity or complexity of PHD increase.

The collection and sharing of PHD is increasingly common, and technological advancements are continuing to enable data to be collected and shared between SUs and HCPs. These findings highlight the opportunities but also the challenges that must be overcome if we are to ensure already overstretched HCPs are not over-burdened, and that these data are used in a way that supports people with HIV to maximise the health and wellbeing across the life course.

### Future recommendations

Our study has identified and described several opportunities for PHD tracking and sharing in the context of HIV care as well as key challenges. It highlights the importance of responding to concerns about data privacy and security as well as finding ways to incorporate PHD into the workflow of a clinic appointment. These issues will be of increased importance and urgency in the context of moves towards further incorporating PHD and data tracking tools into healthcare.^19^

Future research should explore these issues with groups underrepresented in our study, including women, people from racially minoritised backgrounds and those with histories of migration. Additionally, a key focus area should be people living with HIV who are at a higher risk of poor health in order to explore whether PHD might help to facilitate better health outcomes.

## Supplemental Material

sj-docx-1-dhj-10.1177_20552076251383420 - Supplemental material for The challenges and opportunities of personal health data tracking and sharing amongst people living with HIV in the United Kingdom and their specialist healthcare providers Supplemental material, sj-docx-1-dhj-10.1177_20552076251383420 for The challenges and opportunities of personal health data tracking and sharing amongst people living with HIV in the United Kingdom and their specialist healthcare providers by Emily Jay Nicholls, Karen C Lloyd, Katarina Hoernke, Alexander Maddams, Caroline Claisse, Abigail C Durrant, Shema Tariq and Jo Gibbs in DIGITAL HEALTH

sj-docx-2-dhj-10.1177_20552076251383420 - Supplemental material for The challenges and opportunities of personal health data tracking and sharing amongst people living with HIV in the United Kingdom and their specialist healthcare providers Supplemental material, sj-docx-2-dhj-10.1177_20552076251383420 for The challenges and opportunities of personal health data tracking and sharing amongst people living with HIV in the United Kingdom and their specialist healthcare providers by Emily Jay Nicholls, Karen C Lloyd, Katarina Hoernke, Alexander Maddams, Caroline Claisse, Abigail C Durrant, Shema Tariq and Jo Gibbs in DIGITAL HEALTH

sj-docx-3-dhj-10.1177_20552076251383420 - Supplemental material for The challenges and opportunities of personal health data tracking and sharing amongst people living with HIV in the United Kingdom and their specialist healthcare providers Supplemental material, sj-docx-3-dhj-10.1177_20552076251383420 for The challenges and opportunities of personal health data tracking and sharing amongst people living with HIV in the United Kingdom and their specialist healthcare providers by Emily Jay Nicholls, Karen C Lloyd, Katarina Hoernke, Alexander Maddams, Caroline Claisse, Abigail C Durrant, Shema Tariq and Jo Gibbs in DIGITAL HEALTH

sj-docx-4-dhj-10.1177_20552076251383420 - Supplemental material for The challenges and opportunities of personal health data tracking and sharing amongst people living with HIV in the United Kingdom and their specialist healthcare providers Supplemental material, sj-docx-4-dhj-10.1177_20552076251383420 for The challenges and opportunities of personal health data tracking and sharing amongst people living with HIV in the United Kingdom and their specialist healthcare providers by Emily Jay Nicholls, Karen C Lloyd, Katarina Hoernke, Alexander Maddams, Caroline Claisse, Abigail C Durrant, Shema Tariq and Jo Gibbs in DIGITAL HEALTH
