# Supplementary material for: The challenges and opportunities of personal health data tracking and sharing amongst people living with HIV in the United Kingdom and their specialist healthcare providers
Source: Digit Health. 2025 Sep 26;11:20552076251383420. doi: 10.1177/20552076251383420 (PMC12475346; doi:10.1177/20552076251383420)
Supplement: sj-docx-1-dhj-10.1177_20552076251383420 - Supplemental material for The challenges and opportunities of personal health data tracking and sharing amongst people living with HIV in the United Kingdom and their specialist healthcare providers [file sj-docx-1-dhj-10.1177_20552076251383420.docx]

# Coding Tree Service User Interviews

| Theme | Code |
| --- | --- |
| Challenges | Creating unhealthy preoccupation |
|  | Difficult to access clinical data |
|  | HCP doesn't do anything with data shared |
|  | Lack of digital literacy competency |
|  | Limits on data it is possible to record |
|  | Not all data is relevant to care |
|  | Not recording data |
|  | Not sharing data |
|  | Privacy concerns |
|  | Reason for sharing not clear |
|  | Time burden and inconvenience |
| Opportunities | Access to blood results |
|  | Accessibility |
|  | Aiding decision making |
|  | Altruism |
|  | Confirming self-knowledge of health |
|  | Continuity of care |
|  | Convenience |
|  | Creating online one stop shop |
|  | Efficiency |
|  | Emphasis on good and bad information |
|  | Enjoyment |
|  | Identifying patterns |
|  | Improving care |
|  | Interoperability |
|  | It's the future |
|  | Memory aid |
|  | Normalising HIV |
|  | Proof of healthy living |
|  | Seeking reassurance |
|  | Self-managing health |
|  | Sharing with others |
|  | Solving a health issue |
|  |  |

# Coding Tree Healthcare Professionals Interviews

| Theme | Code | |
| --- | --- | --- |
| Access concerns | | |
| App development issues | | |
| Appointments | Challenges | |
|  | Impact of PGData on appointments | |
|  | Priorities | |
|  | Assumption all NHS data is linked in one database | |
| Assumption all NHS data is linked | | |
| Balancing duty of care and confidentiality | | |
| Being health conscious | | |
| Challenges of personal health data sharing | Boundaries | |
|  | Commercialisation of data | |
|  | Competing priorities | |
|  | Concerns about PGdata sharing | |
|  | Data overload | |
|  | Data shared not relevant | |
|  | Digital inequalities | |
|  | Lack of engagement in data sharing | |
|  | No consent to share data with other HCPs | |
|  | Preferences for dialogue | |
|  | Privacy concerns | |
|  | Security concerns | |
|  | Stigma | |
|  | Technological barriers to PGData sharing | |
|  | Technology overload | |
|  | Time burden for service users | |
|  | Time burden HCPs | |
|  | Time constraints | |
|  | Trust in reliability of self-captured data | |
|  | Worry or fixation on data | |
|  |  | |
|  |  | |
| Consent | | |
| COVID related challenges | | |
| Data recording methods | App | |
|  | Computer | |
|  | Material artefact | |
|  | Medical records | |
|  | Paper diary | |
|  | Phone | |
|  | Photos | |
|  | Questionnaire | |
|  | Recall | |
|  | Wearable device | |
| Data requested by HCP | | |
| Data sharing and communication GPs | | |
| Data sharing and communication other HCPs | | |
| Data sharing and communication within HIV Team | | |
| Data sharing methods HCP to SU | | |
| Data sharing methods SU to HCP | | |
| Data types | Adherence | |
|  | Alcohol | |
|  | Allergies | |
|  | Blood pressure | |
|  | Blood results | |
|  | Blood sugar | |
|  | CD4 | |
|  | Cholesterol | |
|  | Contraception and pregnancy | |
|  | Daily practices | |
|  | Date of birth | |
|  | Diet | |
|  | Documents related to social needs | |
|  | Domestic violence | |
|  | Family status | |
|  | Financial | |
|  | Fitness or step counts | |
|  | General wellbeing | |
|  | Health risk assessments | |
|  | Height | |
|  | HIV related complications | |
|  | HIV status | |
|  | Medical records other specialties | |
|  | Medications | |
|  | Menstrual cycle | |
|  | Mood | |
|  | Name | |
|  | Other LTCs | |
|  | Pain | |
|  | Prompts for discussion | |
|  | Recreational drug use | |
|  | Relationships | |
|  | Sexual behaviour | |
|  | Sexual health screening | |
|  | Sexual partners | |
|  | Sleep | |
|  | Smear tests | |
|  | Smoking | |
|  | STIs | |
|  | Symptoms or side effects | |
|  | Temperature | |
|  | Travel history | |
|  | Urine or poo | |
|  | Vaccination record | |
|  | Viral load | |
|  | Weight | |
| Data visualisations | Annotations | |
|  | Mood | |
|  | Sleep | Bar chart |
|  |  | Line graph |
|  |  | Table |
|  |  | Time frame |
| Desire for linked up systems with primary care | | |
| Differing interest levels in health data | | |
| Existing electronic patient record | | |
| Experience of PGData sharing in consultations | | |
| GPs - relationship and communication with | | |
| GPs meant to manage non-HIV health | | |
| HIV care changes | | |
| HIV clinic as one stop shop | | |
| HIV journey | | |
| Ideal tracking and sharing practices | | |
| Information shared by HCP | Alcohol | |
|  | Form for sharing | |
|  | Medication | |
|  | Menopause | |
|  | Recreational drug use | |
|  | Sexual health | |
|  | Smoking | |
|  | U=U | |
| Information sharing from HCP to SU | | |
| Methods for sharing with HIV team | | |
| Monitoring done by GPs or other specialists | | |
| Opportunities for personal health data sharing | Big Data | |
|  | Bonus not replacement for traditional care | |
|  | Continuity of care - changing HCPs or clinics | |
|  | Data accessability | |
|  | Efficiency | |
|  | Holistic approach to care | |
|  | Normalising HIV | |
|  | Ownership or autonomy over own health | |
|  | Prioritising service user needs | |
|  | Reassurance | |
|  | Reliability of self-tracked data | |
|  | PGdata used as... | Facilitating communication |
|  |  | For goal setting |
|  |  | For problem solving |
|  |  | For showing change over time |
|  |  | PGdata as a prompt |
|  |  | PGData as action point to encourage change |
|  |  | PGData as memory aid |
|  |  | Self-reflection |
| Professional background | | |
| Reasons for NOT self-tracking | | |
| Reasons for Self Tracking | | |
| Recording for themselves but not sharing | | |
| Relationships between HIV team and SU | | |
| Relationships with other specialists | | |
| Relevance of data shared to context | | |
| Security | | |
| Stigma | | |
| Temporality of Data Sharing | | |
| Third party data sharing | | |
| Time burden of self tracking | | |
| Trust in PGData | | |
| Trust of GPs | | |
| Who would use an PGdata app | | |
| Worry or fixation on data | | |
