# Supplementary material for: The challenges and opportunities of personal health data tracking and sharing amongst people living with HIV in the United Kingdom and their specialist healthcare providers
Source: Digit Health. 2025 Sep 26;11:20552076251383420. doi: 10.1177/20552076251383420 (PMC12475346; doi:10.1177/20552076251383420)
Supplement: sj-docx-2-dhj-10.1177_20552076251383420 - Supplemental material for The challenges and opportunities of personal health data tracking and sharing amongst people living with HIV in the United Kingdom and their specialist healthcare providers [file sj-docx-2-dhj-10.1177_20552076251383420.docx]

**Semi Structured Interview: HCP Topic Guide**

**Section 1: Background**

**Q1 Please tell us about your experience of working in HIV medicine?**

- When did you first start?
- What is your current role?
- How have things changed?
- What are your main concerns about HIV care at the moment?

**Section 2: Current clinical care**

**Q2 What are your HIV clinic appointments like?**

- How often are patients followed up?
- How long are the appointments?
- Who do you see?
- How do you feel about these appointments?
- Have they changed since you first started working in HIV medicine?
- Has the COVID-19 pandemic changed the way you do these appointments?
- If so, how?

**Q3 What do you need to accomplish in your consultations?**

- What are your priorities?
- What are the current challenges?

**Q4 What information is important for patients to receive during your appointments?**

- How is that information usually provided?
- Is there anything you usually give patients to take away from your consultations?
- Are there any ways you think information sharing between you and your patients could be improved?

**Section 3: Collecting information**

**Q5 What, if any, data do your patients already collect and share about their health and lifestyle?**

- - What, if any, data do they collect about their HIV?
- What, if any, data do they collect about other long-term conditions?
- What, if any, data do they collect about any other aspects of their health?
- Do you ask them to collect these or other kinds of data?
- Is there any other information that you would like them to collect? Why?
  - Has COVID-19 changed the kinds of information that your patients are collecting? If so, how?
  - Why do you think this is?

**Q6 How do they collect this data?**

- - What materials or technologies do they use for collecting this data?
  - Has COVID-19 changed how people are collecting this data? If so, how?

Why do you think this is?

**Q7 Why do you think they collect this data?**

- - Probe for reasons for collecting specific kinds of data already discussed.

**Q8 How do they share this data with you during your consultations with them?**

- - Do they bring anything to their appointments in order to share this data? (e.g. a paper diary, print outs from the computer, their mobile phones, etc.)
  - Is this a useful way for them to share this information with you? If so, why? If not, what would work better?
  - Has the way that patients share information with your or the HIV team changed as a result of COVID-19? If so, in what ways?

**Q9 What is it like to have them share this data with you?**

- - How does it affect your consultations with them? How do you use this information?
  - Is there a format that would be particularly helpful for you to receive this information in?
  - How do you balance sharing data with your patients and other priorities you have for your consultations?
  - What happens if patients share information with you that isn’t helpful for your consultations? (How do they deal with this? Do they feel able to signpost them to other resources or other providers where that information might be useful to share?)

**Q10 Who else do you think it would be helpful for patients to share this information with?**

- Probe for different kinds of data that could be shared and different sharing contexts/relationships.

**Section 4: Future developments**

We are interested in how patient generated data could be used to improve the care of HIV and other long-term conditions. Patient generated data is information that patients collect about themselves such as symptoms, how often they take medication. Now I would like to ask you some questions about what you think about your patients collecting various kinds of data about their health and their lifestyle and how comfortable you are with this data being shared with others.

**Q11 What kind of information, if any, do you think would be useful for patients to collect themselves?**

- About their HIV?
- About other long-term conditions they may be living with?
- About other aspects of their health?

**Q12 Would you like them to share this data with you and or/ the wider HIV team?**

- If so, how?
- Why is it important?

**Q13 Do you think changes to the HIV service [note any they have mentioned] as a result of COVID-19 will change the kinds of data that it would be helpful to have patients collect about themselves in the future?**

- If so, how?
- How do you feel about these changes?

Has it changed the ways that you would like them to share this data with you? If so, how?

**Q14 There are many third party organisations who use patient data to conduct research, develop new medicines and devices, or make funding decisions. How would you feel about your patient’s personal information being shared with others outside of the clinic, such as tech companies, pharmaceutical companies, universities doing research, or HIV charities, with their permission?**

- Is there information you would feel comfortable sharing with these kinds of organisations if you knew it was fully anonymous and your patient had given their permission?
- Is there information you would not feel comfortable sharing at all? Why do you feel this way?
- Are there organisations you would feel more comfortable sharing this information with than others?
- How do you decide what kinds of organisations you could trust to handle your patients’ information securely and in a way that protects their privacy?

**Q15 How do you think digital technology could help them collect and share this data? This includes online diaries, mobile apps or wearable devices, like a Fitbit.**

- Do you think your patients would want to use any of these technologies to record and share their personal information?
- Is there anything else that would be better for collecting and sharing this information – even something that doesn’t exist yet but you would like it to?

**Q16 Our study team is interested in developing technology, like a website or an app that could be used on a mobile phone, so that people living with HIV could collect information about their health or their lifestyle and could also chose to share some of that information directly with their healthcare providers or others that they choose. We have created an initial idea of what we think the website or app may look like [shows prototype to participant].**

- How could this this be beneficial to the care you provide your patients?
- What kind of things would you like it to help your patients do?
- What features do you think it would be helpful for it to have?
- What do you think of the design?
- Is there anything that you think we should change or add?

**Section 5: Rounding up**

**Q17 How has the interview been for you today?**

**Q18 Do you have anything else you would like to say before we finish?**

**Ending the session**

**Thank you for taking the time to talk with me today.**
