# Supplementary material for: The challenges and opportunities of personal health data tracking and sharing amongst people living with HIV in the United Kingdom and their specialist healthcare providers
Source: Digit Health. 2025 Sep 26;11:20552076251383420. doi: 10.1177/20552076251383420 (PMC12475346; doi:10.1177/20552076251383420)
Supplement: sj-docx-3-dhj-10.1177_20552076251383420 - Supplemental material for The challenges and opportunities of personal health data tracking and sharing amongst people living with HIV in the United Kingdom and their specialist healthcare providers [file sj-docx-3-dhj-10.1177_20552076251383420.docx]

**Semi Structured Interview: Service User Topic Guide**

**Section 1: Background**

**Q1 Please tell us about your journey since you first learned about your HIV status?**

- When did you first learn you were living with HIV?
- How has it been since then?
- How have things changed?
- What are your main concerns at the moment?

**Section 2: Current clinical care**

**Q2 What are your appointments with the HIV clinic like?**

- How often do you have clinic appointments?
- How long are the appointments?
- Who do you see or talk to?
- How do you feel about these appointments?
- Have they changed since you first learned about your HIV status?
- Is there anything you usually bring with you to your appointments?
- Has the coronavirus pandemic changed the care you receive at the clinic?
- If so, how? Probe for the ways that it has changed care.

**Q3 What information is important for you to share when you have your clinic appointment?**

- What is it you want the healthcare professional to know?
- How do you usually share this?
- Is there anything you do to prepare this information to share before you have your appointment?

Why is it important for you to share this information during your appointment?

**Q4 What information is important for you to receive when you have your appointment?**

- Is there any information that you think is important to take away from your appointment?
- How is that usually provided to you?

**Section 3: Collecting information**

**Q5 What, if any, information do you already collect about your health and lifestyle?**

- - What, if any, information do you already collect about your HIV?
  - What, if any, information do you already collect about other health conditions?
  - Do you collect information about anything else that you haven’t already mentioned?
  - Is there any other information that you would like to collect?
  - Has the coronavirus pandemic changed the kinds of information that you collect about your health? If so, how?
  - Why do you think it has changed the kinds of information you collect?

**Q6 How do you collect this information?**

- How often do you collect this information?
- How do you record this information? (e.g. materials, in a paper diary or booklet, on their mobile, on a computer, using the internet, etc.)
- What is it like to collect this information about yourself?
  - - What is it like to collect day to day?
    - What has it been like to collect over time? Has anything changed?
  - Have you changed how you collect this information due to the changes that have happened during the coronavirus pandemic?

**Q7 Why do you collect this information?**

- Probe about reasons for collecting each specific kind of information discussed above.
- Why did you first begin collecting this information? Why have you continued to collect it?
- Has this changed recently?

**Q8 Who do you share this information with?**

- Why is it important for you to share this information with them?
- How do you share this information with them?
- What is it like to share this information?
- For clinical contexts, “How has your healthcare provider responded to your sharing of this information with them?”
- Has the coronavirus pandemic changed the way you share information or communicate with your HIV care team? If so, how?

**Q9 Who would you like to share some or all of this information with?**

- Probe for each kind of information and for different sharing contexts/relationships (e.g. to healthcare providers, friends/family, partners, peers including those you may know only online, etc.)
- Why would like you to share this with them?

**Section 4: Future developments**

We are interested in how the information that people living with HIV may collect and share about themselves can be used to improve the care of HIV and other long-term conditions. Now I would like to ask you some questions about what you think about collecting information about your health and lifestyle and your feelings about sharing it with others.

**Q10 What kinds of information, if any, do you think would be useful to collect yourself?**

- - About your HIV?
  - About any other long-term conditions?
  - About any other aspects of your health and wellbeing?

**Q11 Would you like to share this information with your HIV team?**

- If so, why is it important to share this?
- If so, how?

**Q12 How would you feel about this information being shared with others outside of the clinic, such as pharmaceutical companies, universities doing research, or HIV charities?**

- Is there information you would feel comfortable sharing with these kinds of organisations if you knew it was fully anonymous (they wouldn’t find out your name or any other identifying information about you)?
- Is there information you would not feel comfortable sharing at all?
- Why do you feel this way? Are there organisations you would feel more comfortable sharing your information with than others?
- How do you decide what kinds of organisations you could trust to handle your information securely and in a way that protects your privacy?

**Q13 Would you want to use digital health tools, like an online diary, an app you use on your mobile phone, or a Fitbit, to record and share information about your health?**

- How do you think this technology could help you collect and share this information?
- Is there anything else that would be better for collecting and sharing this information – even something that doesn’t exist yet but you would like it to?

**Q14 Our study team is thinking about developing a website or an app that could be used on a mobile phone, so that people living with HIV could collect information about their health or their lifestyle and could also chose to share some of this information directly with their healthcare providers or with others. We have created an initial idea of what we think the website or app may look like [shows prototype to participant].**

- What do you think about using something like this?
- What would you like it to be able to help you do?
- Is there anything that you think we should change or add?
- What do you think about how it looks?
- Would you prefer to have it actually on your phone, or accessible via a web-browser, or both?
- What would it need to do to assure you than your information was kept securely and only shared with those you wish to share it with?

**Section 5: Rounding up**

**Q15 How has the interview been for you today?**

**Q16 Do you have anything else you would like to say before we finish?**

**Ending the session**

**Thank you so much for taking the time to talk with me today.**
