# Supplementary material for: The challenges and opportunities of personal health data tracking and sharing amongst people living with HIV in the United Kingdom and their specialist healthcare providers
Source: Digit Health. 2025 Sep 26;11:20552076251383420. doi: 10.1177/20552076251383420 (PMC12475346; doi:10.1177/20552076251383420)
Supplement: sj-docx-4-dhj-10.1177_20552076251383420 - Supplemental material for The challenges and opportunities of personal health data tracking and sharing amongst people living with HIV in the United Kingdom and their specialist healthcare providers [file sj-docx-4-dhj-10.1177_20552076251383420.docx]

**COREQ 32-item checklist**

For further information about the COREQ guidelines, please see Tong *et al.*, 2017: <https://doi.org/10.1093/intqhc/mzm042>

| **Topic** | **Item No.** | **Guide Questions/Description** | **Page number** |
| --- | --- | --- | --- |
| **Domain 1: Research team and reflexivity** | | |  |
| *Personal characteristics* | | |  |
| Interviewer/facilitator | 1 | Which author/s conducted the interview or focus group? | Pg 6 |
| Credentials | 2 | What were the researcher’s credentials? E.g. PhD, MD | Pg 6 |
| Occupation | 3 | What was their occupation at the time of the study? | Pg 6 |
| Gender | 4 | Was the researcher male or female? | Pg 6 |
| Experience and training | 5 | What experience or training did the researcher have? | Occupation and credentials described pg 5 so some of this is implied. We felt a detailed discussion of our research backgrounds would be an excessive level of detail. |
| *Relationship with participants* | | |  |
| Relationship established | 6 | Was a relationship established prior to study commencement? | Pg 4-5: “Participants were not known to the interviewer before being approached to take part in the study.” |
| Participant knowledge of the interviewer | 7 | What did the participants know about the researcher? e.g. personal goals, reasons for doing the research | Not described. We felt a detailed discussion of this would be an excessive level of detail. |
| Interviewer characteristics | 8 | What characteristics were reported about the inter viewer/facilitator? e.g. Bias, assumptions, reasons and interests in the research topic | Not described. We felt a detailed discussion of this would be an excessive level of detail. |
| **Domain 2: Study design** | | |  |
| *Theoretical framework* | | |  |
| Methodological orientation and Theory | 9 | What methodological orientation was stated to underpin the study? e.g. grounded theory, discourse analysis, ethnography, phenomenology, content analysis | Qualitative health research using thematic analysis (described pg 6). |
| *Participant selection* | | |  |
| Sampling | 10 | How were participants selected? e.g. purposive, convenience, consecutive, snowball | Pg 4-5 (convenience and purposive) |
| Method of approach | 11 | How were participants approached? e.g. face-to-face, telephone, mail, email | Pg 5 |
| Sample size | 12 | How many participants were in the study? | Pg 4 |
| Non-participation | 13 | How many people refused to participate or dropped out? Reasons? | Not reported – it would be unusual to keep track of this for a study of this type. |
| *Setting* | | |  |
| Setting of data collection | 14 | Where was the data collected? e.g. home, clinic, workplace | Pg 5 |
| Presence of non-participants | 15 | Was anyone else present besides the participants and researchers? | This is not reported as there was no one else present and it would be notable if they had been, i.e. we feel it is implied that had other people been present this would have been reported. |
| Description of sample | 16 | What are the important characteristics of the sample? e.g. demographic data, date | Described pg 7 and reported in table uploaded separately. |
| *Data collection* | | |  |
| Interview guide | 17 | Were questions, prompts, guides provided by the authors? Was it pilot tested? | Topic guides uploaded as supplementary files. This was not pilot tested (we would report this if it had been). |
| Repeat interviews | 18 | Were repeat inter views carried out? If yes, how many? | No. |
| Audio/visual recording | 19 | Did the research use audio or visual recording to collect the data? | Yes, reported pg 5 |
| Field notes | 20 | Were field notes made during and/or after the inter view or focus group? | Not reported |
| Duration | 21 | What was the duration of the inter views or focus group? | Pg 5 |
| Data saturation | 22 | Was data saturation discussed? | Pg 6 |
| Transcripts returned | 23 | Were transcripts returned to participants for comment and/or correction | No |
| **Domain 3: analysis and findings** | | |  |
| *Data analysis* | | |  |
| Number of data coders | 24 | How many data coders coded the data? | Pg 6 |
| Description of the coding tree | 25 | Did authors provide a description of the coding tree? | Coding tree uploaded as supplementary file |
| Derivation of themes | 26 | Were themes identified in advance or derived from the data? | Pg 6 |
| Software | 27 | What software, if applicable, was used to manage the data? | Pg 6 |
| Participant checking | 28 | Did participants provide feedback on the findings? | No |
| *Reporting* | | |  |
| Quotations presented | 29 | Were participant quotations presented to illustrate the themes/findings? Was each quotation identified? e.g. participant number | Quotes provided plus demographic information, but participant number not included. Pg 8-13. |
| Data and findings consistent | 30 | Was there consistency between the data presented and the findings? | Pg 8-13 |
| Clarity of major themes | 31 | Were major themes clearly presented in the findings? | Pg 8-13 |
| Clarity of minor themes | 32 | Is there a description of diverse cases or discussion of minor themes? | Pg 8-13 |
